# Global Perspectives on Sleep Health: Definitions, Disparities, and Implications for Public Health

**DOI:** 10.3390/brainsci15030304

**Published:** 2025-03-13

**Authors:** Lourdes M. DelRosso

**Affiliations:** Division of Pulmonary and Sleep Medicine, Department of Medicine, University of California San Francisco, Fresno, CA 93701, USA; lourdes.delrosso@ucsf.edu

**Keywords:** sleep health, sleep disorders, circadian health, disparities

## Abstract

Sleep health is a multidimensional construct encompassing sleep quality, duration, efficiency, regularity, and alignment with circadian rhythms, playing a crucial role in overall well-being. Sleep health remains inconsistently defined across research and clinical settings despite its importance, limiting the ability to standardize assessments and interventions. Recent studies have emphasized the significance of defining sleep health beyond the absence of sleep disorders, integrating subjective and objective measures to assess its impact on physical and mental health outcomes. Disparities in sleep health exist across gender, socioeconomic status, and geographic regions, particularly in low- and middle-income countries where inconsistent work schedules, economic stress, and healthcare access influence sleep patterns. Poor sleep health is associated with increased risks of cardiovascular disease, obesity, metabolic dysfunction, and mental health disorders, reinforcing its role as a modifiable risk factor in public health. Lifestyle factors such as caffeine consumption, physical activity, and irregular eating patterns also contribute to sleep disturbances, highlighting the need for behavioral interventions. This narrative review aims to synthesize the current knowledge on sleep health, focusing on its definitions, measurement tools, global disparities, and associations.

## 1. Introduction

Sleep is a fundamental pillar of health, as vital as nutrition and exercise [1]. It supports cognitive function, immune defense, metabolic regulation, and emotional stability [2,3], directly impacting overall well-being and quality of life [4]. Adequate sleep enhances memory consolidation, decision-making, and emotional resilience, while chronic sleep deprivation is linked to increased risks of cardiovascular disease, obesity, diabetes, and mental health disorders [2]. However, despite its critical role, there is no universally accepted definition of sleep health. While researchers acknowledge that sleep health encompasses both sleep quality and quantity, measuring and standardizing it remains a challenge. This gap in definition complicates efforts to develop effective public health policies and clinical interventions, contributing to the widespread neglect of sleep as a modifiable health factor.

Early studies focused primarily on defining the amount of sleep necessary for maintaining health [5,6]. However, this approach failed to capture the multidimensional nature of sleep health, leading researchers to explore broader conceptual frameworks. One of the most widely recognized definitions comes from Buysse, who proposed a comprehensive, multidimensional definition of sleep health that extends beyond the absence of a sleep disorder [7]. His definition of sleep health includes five dimensions: sleep duration– the total amount of sleep obtained within 24 h; sleep quality—the subjective perception of how restorative and uninterrupted sleep is; sleep timing—the alignment of sleep with circadian rhythms; sleep efficiency—the proportion of time spent asleep relative to time in bed; and sleep regularity—the consistency of sleep and wake patterns over time [7].

Despite the growing adoption of this framework, no universally accepted definition of sleep health existed, leading to inconsistencies in research and clinical assessments. To address this, various organizations have attempted to standardize sleep health metrics. In 2017, the National Sleep Foundation established a panel of experts to assess evidence-based sleep quality indicators across different age groups [8]. Using Delphi consensus guidelines to identify 277 studies; the panel showed that sleep continuity variables (sleep latency, number of awakenings longer than five minutes, wake after sleep onset, and sleep efficiency) were appropriate measures of sleep quality throughout the lifespan, with sleep architecture and napping having a less clear role in defining sleep quality [8]. In 2023, the National Sleep Foundation expanded its framework, adding sleep regularity and awakening to the markers of sleep health [9].

The significance of these parameters is highlighted by research linking the elements of sleep health to chronic disease risk. For example, Nistor et al. conducted a systematic review to examine the interaction and association between some of these parameters, particularly sleep quality and sleep duration, with the prevalence of multimorbidity, which is defined as the occurrence of two or more chronic disorders in adults living independently [10]. The systematic review included 29 studies (24 cross-sectional and 5 prospective cohorts) comprising 481,862 participants from 16 countries. Although the study found that poor sleep quality and sleep durations were associated with a higher prevalence of multimorbidity, there were significant variations in the definitions of the sleep parameters [10].

Despite efforts by researchers and organizations to incorporate physiological and behavioral components into the definition of healthy sleep, there is still no universally accepted definition, making comparing epidemiological data across different populations challenging. Moreover, emerging research suggests that additional parameters, such as restlessness and cyclic alternating pattern, may be contributors to sleep disruptions [11,12]. These findings highlight the need to continuously refine sleep health frameworks to better capture the complexity of sleep across different populations and life stages.

This narrative literature review aims to synthesize current definitions, evaluation methods, and global perspectives on sleep health, recognizing that sleep habits and disorders vary widely depending on cultural, environmental, and other social factors. By consolidating existing knowledge, identifying knowledge gaps, and proposing future research directions, this review seeks to advance the recognition of sleep health as an essential determinant of overall well-being, warranting greater emphasis in clinical practice and public health policies.

## 2. Review

Understanding sleep health requires comprehensively examining its various definitions, assessment methods, and contributing factors across different populations and global regions. This section synthesizes key studies on sleep health, organizing them into three main areas: defining sleep health and measurement tools—exploring different definitions and validated assessment tools; demographic influences on sleep health—examining age- and gender-related differences; and global disparities in sleep health—addressing socioeconomic, cultural, and environmental factors that shape sleep patterns. Studies included in this review are research in the last decade that sought to clarify what constitutes sleep health, how it should be measured, and how it impacts health outcomes. Reviews, opinion statements, editorials, and research on sleep quality in specific medical conditions, medication, or drug effects were not included. Equally, interventions to improve sleep quality are beyond the scope of this review.

### 2.1. Defining Sleep Health and Measurement Tools

The definition of sleep health has evolved significantly, moving beyond a singular focus on sleep duration to encompass a broader, multidimensional framework, including sleep quality, efficiency, regularity, and circadian alignment. Various tools are currently used to aid with the definition of sleep health.

#### 2.1.1. Defining Sleep Health

Historically, sleep duration has been the most commonly used metric to define sleep health. Murawski et al. explored the impact of a mobile health intervention on physical activity among adults [13]. The authors defined sleep health primarily through duration, assessing whether participants met the recommended 7–9 h of sleep per night [13]. The American Academy of Sleep Medicine (AASM) and the Sleep Research Society (SRS) issued a joint consensus statement recommending that adults obtain at least 7 h of sleep per night to maintain optimal health and reduce the risk of adverse outcomes such as cardiovascular disease, obesity, diabetes, and neurocognitive decline [6]. More recent frameworks emphasize that sleep health is not solely determined by duration but rather by a combination of sleep behaviors. One of the most widely cited models is Buysse’s SATED framework, which defines sleep health based on five core dimensions: sleep satisfaction, alertness during waking hours, timing of sleep, sleep efficiency, and sleep duration [7]. Eze et al. (2023) apply the SATED framework to investigate sleep health among neurology outpatients from the Adult Neurology Clinic at Alex Ekwueme Federal University Teaching Hospital in Abakaliki, Nigeria. Each dimension was scored from 0 to 2, with a total score ranging from 0 to 10; 8 to 10 indicated good sleep health, while scores below 8 indicated poor sleep health [14]. The study included 267 patients, revealing that only 19% of participants achieved good sleep health (SATED scores 8–10), whereas 81% experienced poor sleep health [14]. Expanding on SATED, the RU-SATED questionnaire was developed to incorporate an additional component: regularity [15]. The RU-SATED scale has undergone various cross-cultural adaptations and language validations.

Meng et al. (2023) aimed to adapt and validate the RU-SATED questionnaire for assessing sleep health among Chinese healthcare students [16]. The RU-SATED is also validated in Portuguese [17], Spanish [18], French [19], and Japanese [20].

In addition to these models, the National Sleep Foundation (NSF) published an expert consensus definition of sleep health, highlighting sleep continuity, efficiency, awakenings, and architecture as key markers [21]. In 2023, the NSF expanded this framework, adding sleep regularity and awakening consistency as essential components of healthy sleep [9].

#### 2.1.2. Metrics Used to Assess Sleep Health

Researchers employ self-reported surveys, actigraphy-based assessments, and polysomnography to assess sleep health, each offering unique advantages and limitations. One commonly used tool is the NIH Patient-Reported Outcomes Measurement Information System (PROMIS™), which is used to assess the impact of sleep on overall well-being. Kolobaric et al. explored and used PROMIS and the Sleep Disturbance SF 8A as the primary tool to determine sleep health, defining sleep quality, difficulty falling asleep, and overall restfulness [22]. The researchers also considered the interconnectedness of sleep with other health outcomes, including stress, anxiety, pain, and general well-being [22]. Another common tool to assess sleep quality is the Pittsburgh Sleep Quality Index (PSQI), developed by Buysse et al. (1989). It evaluates seven components: subjective sleep quality, sleep latency, sleep duration, habitual sleep efficiency, sleep disturbances, use of sleep medication, and daytime dysfunction. Each component is scored on a scale from 0 to 3, culminating in a global score ranging from 0 to 21; higher scores indicate poorer sleep quality [23]. The PSQI has been used in multiple studies and populations assessing sleep quality [24,25,26]. Purani et al. utilized the PSQI to assess sleep quality among 32 adult smokers over 12 weeks. Their findings indicated that participants had poor sleep quality at baseline (PSQI > 5), which was associated with increased withdrawal symptoms and smoking urges. Notably, increased daily exercise was linked to improved sleep quality over time [27]. The use of sleep diaries is exemplified by Kline et al. (2021), who explored the relationship between sleep health and weight loss outcomes. The authors defined sleep health using a composite measure encompassing five dimensions: regularity, satisfaction, alertness, timing, and efficiency [28]. Bliwise et al. used the Medical Outcomes Study Sleep (MOS-Sleep) questionnaire to assess sleep health as a multidimensional construct. The authors used parameters such as sleep disturbance, which captures the frequency and severity of disruptions in sleep initiation and maintenance, and snoring, which indicates potential obstructive sleep apnea. The authors included other important parameters, such as shortness of breath or headaches during sleep and sleep adequacy, which referred to the individual’s perception of whether their sleep was restorative and sufficient. Furthermore, the study included total sleep duration and daytime somnolence [29].

Besides questionnaires and diaries, studies have used actigraphy, home monitors, and polysomnography. Bowman et al. defined sleep health using a multidimensional approach, assessing six components: sleep efficiency, duration, mid-sleep timing, regularity, alertness, and satisfaction. These components were measured through actigraphy over approximately 29 days and self-reported assessments [30]. Griffith et al. explored the relationship between eating behaviors and sleep health in a cohort of 52 young adults without chronic diseases [31]. The team defined sleep health using wrist actigraphy and sleep diaries over seven days, with parameters such as bedtime, wake time, total sleep time, sleep latency, sleep efficiency, and wake after sleep onset [31]. Wallace et al. aimed to empirically identify distinct domains of sleep health in older adults using actigraphy data. The researchers conducted exploratory factor analyses by analyzing 28 actigraphy-derived sleep measures from 2841 older men and 2719 older women. They identified five consistent sleep health domains across both sexes: timing, efficiency, duration, sleepiness, and regularity [32].

Polysomnography is the gold standard for evaluating various sleep disorders and can aid in identifying normal, healthy sleep [33]. Weibel et al. investigated the impact of consistent caffeine consumption on sleep architecture, mainly focusing on rapid eye movement (REM) sleep using polysomnography values of sleep architecture. The researchers defined sleep health through objective measures, including total sleep time, architecture, REM sleep latency, and subjective interviews [34]. Home sleep testing devices have the potential to track sleep duration, efficiency, and circadian rhythms and aid in the assessment of sleep health. Tam et al. employed the SleepImage Ring^®^ device to objectively measure sleep parameters, including the sleep quality index, arousal index, sleep efficiency, and apneahypopnea index [35]. However, while current devices demonstrate high agreement with polysomnography in detecting sleep–wake patterns, their ability to differentiate sleep stages, arousals, and movements remains limited [36].

### 2.2. Gender and Age Influences on Sleep Health

Beyond definitions and measurement tools, age and gender are crucial in shaping sleep patterns, quality, and overall sleep health. Sleep architecture evolves from childhood to older adulthood, with changes in sleep duration, efficiency, and timing affecting health outcomes [37]. Gender differences in hormonal fluctuations, caregiving responsibilities, and societal expectations further contribute to variations in sleep quality and the prevalence of sleep disorders [38]. Research highlights how sleep disruptions in women often correlate with reproductive milestones (menstruation, pregnancy, and menopause) [39,40,41], whereas men may experience a higher prevalence of sleep-disordered breathing and shorter sleep duration [42]. In aging populations, circadian rhythm alterations and increased sleep fragmentation impact sleep health, leading to greater risks for cognitive decline, metabolic dysfunction, and cardiovascular disease. This section examines sleep health in relation to gender and age, emphasizing the need for age- and sex-specific approaches in sleep assessment and intervention.

#### 2.2.1. Sleep Health in Children and Adolescents

Sleep plays a particularly crucial role in the developing brain. Dadzie et al. investigated the associations between sleep health and child development outcomes, focusing on various sleep parameters and their impact on children’s cognitive, behavioral, and physical development. The study utilized objective sleep measurements, such as actigraphy, alongside parent-reported sleep questionnaires to assess sleep duration, quality, and timing. The findings indicated that adequate sleep duration and quality were positively correlated with better cognitive performance, improved behavioral regulation, and healthier physical growth metrics. Conversely, irregular and insufficient sleep patterns were associated with increased behavioral issues and suboptimal developmental outcomes [43]. Turnbull et al. corroborated these findings in pre-kindergarten children, showing that adequate sleep duration and consistent bedtimes were positively associated with better performance in areas critical to school readiness, such as language development, cognitive functioning, and social–emotional skills [44]. Dong et al. (2019) sought to develop a comprehensive Sleep Health Composite score to evaluate sleep health in adolescents with an evening chronotype. The authors defined sleep health through six dimensions: regularity, satisfaction, alertness, timing, efficiency, and duration. The study involved 176 adolescents, averaging 14.77 years of age, who preferred evening activities and were at increased risk in at least one of five health domains: emotional, cognitive, behavioral, social, or physical. Findings revealed that a higher Sleep Health Composite score, indicating better overall sleep health, was associated with lower risk in emotional, cognitive, and social domains, fewer physical symptoms, and reduced odds of obesity and current mood or anxiety disorders [45].

In terms of tools used in children, Turnbull et al. (2022) utilized a combination of parent-reported surveys, direct child assessments, and teacher evaluations to examine sleep health and its impact on early learning outcomes. Sleep health was assessed through various instruments: The Child and Adolescent Sleep Checklist (CASC) was used to estimate sleep duration and bedtime consistency. The Family Involvement Questionnaire (FIQ) and the Confusion, Hubbub, and Order Scale (CHAOS) were used to evaluate the regularity of bedtime routines and household stability. Various language and cognitive abilities validated questionnaires were used to measure school readiness [44]. Meltzer et al. proposed the Peds B-SATED model as an adaptation of Buysse’s SATED framework for pediatric sleep health, emphasizing the need to consider sleep-related behaviors (B), recognizing that children’s sleep is heavily influenced by bedtime routines, screen exposure, parental influence, and caffeine use [46]. Galand et al. used actigraphy to demonstrate that children’s sleep can benefit from earlier bedtimes, highlighting sleep timing as a critical factor in pediatric sleep health. Their study examined children aged 5 to 12 years and found that those who initially obtained less than 8.5 h of sleep per night experienced a significant increase in total sleep duration when maintaining earlier bedtimes over a week. In contrast, children already sleeping more than 8.5 h per night showed minimal changes in sleep duration. These findings emphasize that bedtime timing is crucial in optimizing sleep health in children, particularly for those who do not meet recommended sleep duration guidelines per age [47].

#### 2.2.2. Sleep Health in Women and Older Adults

While women generally exhibit longer sleep durations, shorter sleep onset latency, and higher sleep efficiency compared to men, they report more sleep disturbances, including insomnia, non-restorative sleep, and increased sleep complaints despite objectively better sleep metrics [48] Studies on women have shown that depressive symptoms have been associated with diminished sleep time in midlife women [30]. Tam et al. revealed that women over 50 experienced a more pronounced decline in sleep quality compared to men, characterized by increased arousal index and apnea–hypopnea index, alongside a decrease in sleep quality index [35].

In terms of amount of sleep, a study in Nigeria showed the average sleep duration was 7.5 ± 1.5 h, with males averaging 7.6 h and females 7.3 h. Patients aged 18–64 averaged 7.4 h, while those aged 65 and above averaged 7.9 h. Among the SATED dimensions, sleep timing had the lowest mean score (0.97), indicating it was the most problematic area. Meanwhile, sleep satisfaction had the highest mean score (1.54), suggesting it was the least affected [14]. In older populations, sleep architecture changes significantly, with aging being associated with shorter total sleep time, reduced sleep efficiency, and increased nighttime awakenings, though these changes tend to be more pronounced in men, who experience a greater reduction in slow-wave sleep compared to women [49]. Interestingly, older adults often report better subjective sleep quality despite objective measures indicating increased sleep fragmentation and wakefulness after sleep onset [50].

### 2.3. Global and Social Determinants of Sleep Health

Sleep health is shaped not only by biological and behavioral factors but also by cultural, socioeconomic, and environmental influences that vary across populations [51]. Research has shown that social and environmental factors can significantly affect sleep duration, quality, and consistency [52]. Studies have also demonstrated that geographical location and climate contribute to disparities in sleep health, particularly in underserved populations [53]. Understanding these global and social determinants is essential for developing tailored interventions and public health policies that promote equitable sleep health access across diverse populations. The following sections explore the role of cultural and socioeconomic disparities, behavioral and environmental influences, and broader social determinants in shaping sleep health outcomes worldwide.

#### 2.3.1. Cultural and Socioeconomic Disparities

In a review protocol by Olorunmoteni, adolescent sleep health in Africa was defined as a multidimensional pattern of sleep–wakefulness that adapts to individual, social, and environmental demands to promote physical and mental well-being [54]. The authors stated that key sleep parameters that assess sleep health include sleep duration, quality, chronotype, and bedtime variability and examined their association with mental health disorders, cardiometabolic risks, and disparities between rural and urban populations [54]. This study integrated the patient’s geographic location into healthy sleep by comparing sleep health between rural and urban populations. Another study found that cultural traditions, such as bedtime rituals and communal sleeping arrangements, contributed to healthier sleep patterns. King et al. used a qualitative methodology, conducting focus groups and individual interviews with 59 American Indian adults aged 30–79 from three geographic locations (Arizona, South Dakota, and Oklahoma) [55]. Twelve facilitators of healthy sleep were identified, with protective cultural factors playing a key role, including praying, storytelling, ceremonies, cultural bedtime routines, and traditional remedies [55]. Participants also highlighted good dreams as a guiding force in their lives, reinforcing the connection between spirituality and sleep quality. Conversely, 11 barriers to healthy sleep were recognized, including poor sleep environments, psychological stress, underlying medical conditions, substance use, nightmares, occupational demands, restlessness, technology use, inconsistent sleep schedules, economic stressors, and dietary factors [55]. A similar study using focus groups to identify facilitators and barriers among Latino populations in 12 different nationalities identified key facilitators of healthy sleep as awareness of sleep hygiene techniques, high motivation to improve sleep for better health, and interest in interventions such as consumer sleep trackers and educational programs [56]. However, barriers to good sleep were prevalent and included stress related to work, family obligations, financial concerns, acculturation, and environmental factors such as noisy sleep environments and shift work schedules [56].

#### 2.3.2. Behavioral and Environmental Factors

Diet, exercise, and use of drugs are important factors that contribute to the definition of sleep health.

Eze et al. showed significant associations between poor sleep health and factors such as smoking, absence of alcohol abuse, and having a neurological diagnosis, with no significant associations with sex or age [14].

Griffith et al. found that the timing of eating significantly influenced sleep–wake patterns and sleep efficiency. Participants who engaged in later eating patterns, including skipping breakfast and consuming food at night, tended to have later bedtime and wake times, experiencing lower sleep efficiency than those with earlier eating patterns who consumed breakfast and avoided nighttime eating (late eaters, 77.0%; early eaters, 84.6%). The duration of eating did not significantly impact sleep health indices [31]. Regular daytime caffeine consumption disrupted REM sleep regulation, leading to delayed REM sleep promotion and diminished sleep quality [34].

Torres-Lopez et al. (2024) investigated the impact of a 20-week exercise training program on sleep parameters in children aged 8 to 11 years with overweight or obesity. The study defined sleep health primarily through objective measures obtained via wrist-worn actigraphy, focusing on metrics such as wake after sleep onset. The study concludes that regular exercise can enhance certain aspects of sleep health, mainly by reducing nocturnal awakenings in children with overweight or obesity [57].

#### 2.3.3. Other Social Determinants of Sleep Health

An important contribution from Madhavan et al. identifies sleep health among migrants from Africa, finding that migrant parents who live with all their young children experience better sleep health compared to those whose children live elsewhere or those without young children [58]. The findings suggest that while parenting can be stressful, the psychological distress of separation from young children may contribute more significantly to sleep problems [58]. Among 3841 female adolescents in Uganda, 15.1% reported poor sleep quality, and 21.6% experienced tiredness [59]. Poor sleep was associated with lower socioeconomic status, smaller household size, and inadequate nutrition, highlighting the influence of social determinants on sleep health [59]. Additionally, poor sleep correlated with menstrual health issues, including increased menstrual pain (AOR = 1.74) [59].

Simonelli et al. (2018) examined the epidemiology of sleep health in low- and middle-income countries by analyzing the prevalence of poor sleep quality and abnormal sleep duration. The authors defined sleep health using Buysse’s five-dimension framework, which includes sleep satisfaction, alertness, timing, efficiency, and duration. The study reviewed 45 publications [60], from China and Brazil, to assess sleep health parameters. The meta-analysis revealed substantial variability in sleep health parameters. Still, poor sleep quality was highly prevalent, with estimates ranging from 6% to 94% in adults and 2% to 86% in older adults, with a pooled prevalence of approximately 33% across all studies. Similarly, self-reported sleep duration ranged between 7 and 8 h per night. However, there was significant variability in study designs, assessment tools, and population characteristics [60]. Etindele Sosso et al. explored the relationship between socioeconomic status and sleep health in African populations, analyzing 43 observational studies that included 153,372 participants from 26 African countries. The study defined sleep health using Buysse’s multidimensional framework, which considers sleep quality, quantity, and the presence of sleep disorders. Socioeconomic status was assessed through education, income, and employment, while sleep health outcomes included sleep duration, insomnia, sleep disturbances, and composite sleep quality measures. The meta-analysis found that low educational attainment was significantly associated with a higher likelihood of insomnia (OR = 1.53) and poor sleep quality (OR = 1.60). At the same time, it was paradoxically linked to longer sleep duration (OR = 0.65). Similarly, low income/assets increased the risk of insomnia (OR = 1.38), and lower occupational status was linked to lower odds of short sleep (OR = 0.49). These findings contribute to the definition of sleep health while acknowledging that significant socioeconomic disparities in sleep health, unemployment, or unstable work conditions can adversely affect sleep [61].

## 3. Discussion

The concept of sleep health has evolved from a simple measure of sleep to a multidimensional framework incorporating quality, duration, efficiency, regularity, and alignment with circadian rhythms but also includes the absence of disease, cultural practices, and a stable socioeconomic status. While substantial progress has been made in defining and measuring sleep health, there are still ongoing challenges in standardization, disparities in sleep health across populations, and integrating sleep health into public health policies.

**Figure 1:**
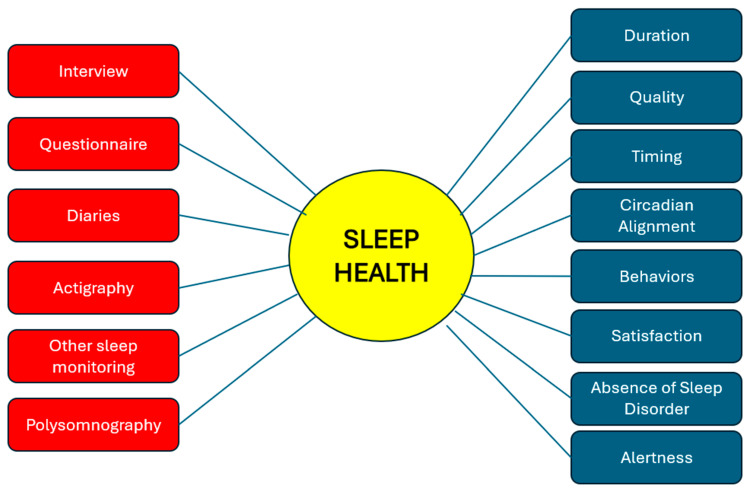
Methods for assessing sleep health and its multidimensional component.

A major barrier to advancing sleep health research is the lack of a universally accepted definition, which complicates cross-population comparisons and limits the ability to implement effective interventions globally. The RU_SATED and SATED scales provide multidimensional tools to evaluate sleep health across populations [16]. However, objective measures such as actigraphy and polysomnography remain underutilized in population-based studies, especially in low-income settings. A systematic review by Simonelli et al. highlighted the significant variability in sleep health assessment tools and definitions, making cross-cultural comparisons challenging [60]. Figure 1 identifies the dimensions of sleep health and the assessment tools.

Significant sleep health disparities exist by gender, age, and socioeconomic status. Findings by Tam et al. (2024) and Bowman et al. demonstrate apparent gender-based differences in sleep health, with women, particularly postmenopausal women, exhibiting higher rates of sleep fragmentation, lower sleep efficiency, and increased prevalence of insomnia [30,35]. Poor sleep health is consistently linked to adverse health outcomes, including increased risk of cardiovascular disease, obesity, metabolic dysfunction, and mental health conditions. Findings from Yan et al. demonstrated that higher sleep fragmentation and lower sleep efficiency were associated with a greater incidence of congestive heart failure [62]. Similarly, the relationship between sleep and obesity was evidenced by Kline et al., who found that better sleep health correlates with improved weight loss outcomes [28].

Behavioral and lifestyle factors also play a significant role in sleep health. Griffith et al. (2024) found that irregular eating patterns, particularly nighttime eating, were associated with poorer sleep efficiency and delayed sleep timing [31]. Similarly, other lifestyle factors such as physical activity, caffeine consumption, and electronic device use play a crucial role in shaping sleep health [34,57].

At a global level, this review highlights the need for sleep health to be integrated into public health policies, particularly in lower-income countries where economic instability, healthcare access limitations, and environmental stressors may compound sleep disturbances. The review by Simonelli et al. (2018) demonstrated that poor sleep health is highly prevalent in low- and middle-income countries, yet sleep research in these regions remains underdeveloped [60]. Given the impact of sleep disturbances on productivity, cognitive performance, and chronic disease risk, public health initiatives should prioritize education, awareness, and access to sleep interventions.

Other reviews have examined the concept of sleep health. Vorster et al. provide a review of its role in brain, metabolic, cardiovascular, and social health, emphasizing its significance alongside other key health behaviors such as diet and exercise. Their work introduces the Bernese Sleep Health Questionnaire, a clinical tool designed to assess sleep health and screen for sleep–wake circadian disorders [63]. While their approach is clinically oriented, the current review expands upon the discussion by addressing the broader public health implications of sleep health, particularly in the context of global, socioeconomic, and cultural influences, integrating multiple definitions from different frameworks and emphasizing the need for standardized assessments. Hale et al. present the relationship between sleep and sleep disorders and the role of sleep as a key health indicator. They argue that poor sleep is both a consequence and a predictor of chronic conditions, including cardiovascular disease (CVD), obesity, mental health disorders, and neurodegenerative diseases [64]. The authors highlight the paradigm shift in sleep research from a disease-centric approach to a preventative health perspective, stressing that poor sleep should be viewed as a risk factor for chronic disease rather than simply a symptom of illness [64]. Meltzer et al. highlight the need for pediatric-specific sleep health measures and call for integrating sleep health assessments into clinical practice, public health initiatives, and research [46].

Recognizing sleep health as a fundamental public health priority will facilitate the development of targeted interventions, awareness campaigns, and policy initiatives aimed at improving sleep health across diverse populations. Integrating sleep education into medical training, launching public awareness campaigns, and adapting sleep recommendations to cultural, social, gender-specific, and age-specific needs will be critical in addressing global sleep disparities and fostering better sleep practices worldwide. Furthermore, policymakers must prioritize sleep health in public health agendas, by funding large-scale longitudinal studies and policy-driven initiatives that address socioeconomic and demographic disparities in sleep health.

Future research should explore the following: standardized sleep health assessment tools that integrate both subjective and objective sleep measures, affordable, accessible sleep interventions for underserved communities, and assessing the impact of education policies on population sleep health.

By implementing multidisciplinary strategies—enhancing sleep medicine training, launching public education campaigns, and adapting sleep guidelines to cultural, gender- related, and age-related differences—sleep health can be elevated to the same level of priority as nutrition and exercise in global public health initiatives.

## 4. Conclusions

Sleep health is a dynamic and multidimensional construct that extends beyond individual sleep duration and quality to encompass cultural, social, gender-related, and age-related factors.

As shown in this review, one of the primary challenges in establishing a universal definition of sleep health is the complex interplay between sleep metrics, all of which are influenced by biological, environmental, and behavioral factors. The widely recommended 7–9 h of sleep per night for adults serves as a general guideline but does not account for individual differences, such as habitual short sleepers (<6 h) who function optimally or long sleepers (>9 h) who require extended sleep duration to maintain well-being. Moreover, sleep quality is inherently more difficult to quantify, as it encompasses subjective factors such as sleep satisfaction, depth, and restfulness.

**Figure 2:**
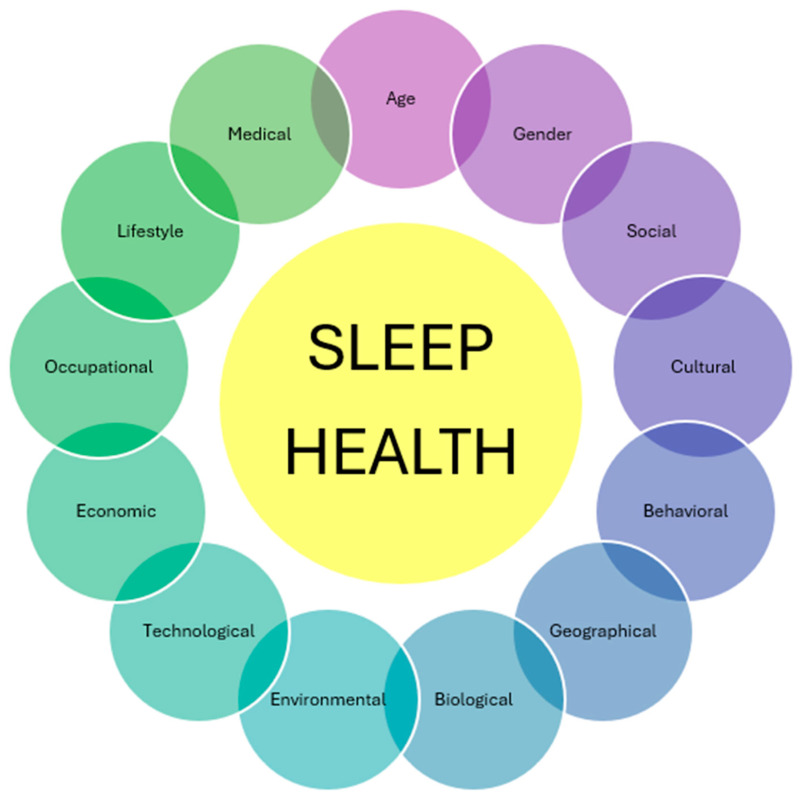
Comprehensive factors affecting sleep health: the interplay of global influences.

Across different global populations, disparities in sleep health arise from socioeconomic conditions, occupational demands, environmental stressors, and access to healthcare, highlighting the need for equitable interventions. Gender-specific differences, including hormonal influences, age-related changes in sleep patterns, and various social practices, demonstrate the need for precision considerations in sleep health policies. Recognizing sleep as a crucial determinant of overall well-being, public health initiatives must integrate culturally sensitive, socially informed, and scientifically validated approaches to promote sleep health across diverse populations. Figure 2 summarizes all the aspects that can influence sleep health in individuals globally.

Future research can incorporate machine learning and wearable technology to further enhance longitudinal tracking, enabling personalized and population-level assessment of sleep health. Furthermore, a precision-based approach to sleep health should focus on refining personalized recommendations that go beyond the one-size-fits-all duration model by integrating factors such as age, genetic predisposition, lifestyle, circadian alignment, and cultural and social determinants.
